# Claims Data Analysis on the Dispensing of Tricyclic Antidepressants Among Patients With Dementia in Germany

**DOI:** 10.3389/fphar.2019.00841

**Published:** 2019-07-24

**Authors:** Philipp Hessmann, Jan Zeidler, Jona Stahmeyer, Sveja Eberhard, Jonathan Vogelgsang, Mona Abdel-Hamid, Claus Wolff-Menzler, Jens Wiltfang, Bernhard Kis

**Affiliations:** ^1^Department of Psychiatry and Psychotherapy, University Medical Center Goettingen, Goettingen, Germany; ^2^Center for Health Economics Research Hannover (CHERH), Leibniz University Hannover, Hannover, Germany; ^3^Health Services Research Unit, AOK Niedersachsen, Hannover, Germany; ^4^German Center for Neurodegenerative Diseases (DZNE), Goettingen, Germany; ^5^iBiMED, Medical Science Department, University of Aveiro, Aveiro, Portugal

**Keywords:** antidepressants, tricyclic, claims data, dementia, pharmacotherapy

## Abstract

**Objective:** A restrictive use of tricyclic antidepressants (TCA) in patients with dementia (PwD) is recommended due to the hazard of anticholinergic side effects. We evaluated the frequency of TCA dispensing in PwD over a period of 1 year and the use of TCA before and after the incident diagnosis of dementia.

**Methods:** This analysis was based on administrative data from a German statutory health insurance for a period of 2 years. Totally, 20,357 patients with an incident diagnosis of dementia in 2014 were included. We evaluated the dispensing of TCA in 2015. Subgroup analyses were conducted to evaluate associations between the incident diagnosis of dementia and modifications in TCA dispensing.

**Results:** In 2015, 1,125 dementia patients (5.5%) were treated with TCA and 31% were medicated with TCA in all four quarters of 2015. Most dispensings were conducted by general practitioners (67.9%). On average, patients received 3.7 ± 2.6 dispensings per year. Amitriptyline (56.3%), doxepin (26.8%), and trimipramine (16.8%) were dispensed most often. Subgroup analyses revealed that the dispensing of TCA remained mainly unchanged following the incident diagnosis.

**Conclusion:** A relevant number of PwD were treated with TCA. To maintain the patients’ safety, an improved implementation of guidelines for the pharmaceutical treatment of PwD in healthcare institutions might be required. Since 68% of the patients suffered from depression, future studies should further evaluate the indications for TCA.

## Introduction

Patients with dementia (PwD) frequently experience comorbid psychiatric disorders like depression, anxiety, and sleep disturbances (Enache et al., 2011; Mortamais et al., 2018). Randomized controlled trials suggest that antidepressants can be effective in treating depression in PwD, although the evidence is inconclusive and there is no evidence of superior efficacy of any particular antidepressant (Dudas et al., 2018). Certain antidepressants can also be used for symptoms like sleep disturbances, anxiety, and restlessness (McCurry et al., 2005). However, the German guideline for the treatment of PwD does not contain specific pharmacological recommendations for the treatment of comorbid sleep disorders or anxiety in PwD (Deuschl and Maier, 2016).

From a neurochemical perspective, the bioavailability of several neurotransmitters is diminished in PwD. This is especially apparent in Alzheimer’s disease (AD), where the reduced availability of acetylcholine and consequent dysfunctions of the cholinergic system are considered essential factors in the occurrence of typical AD symptoms (Pinto et al., 2011). Unfortunately, anticholinergic side effects are often seen in patients using tricyclic antidepressants (TCAs), leading to an increased risk of further cognitive decline, tachycardia, epileptic seizures, delirium, and urinary retention (Patel et al., 2017). TCAs might also increase the risk of impaired coordination and fall due to their sedative effects (O’Neil et al., 2018). Current guidelines therefore recommend a restrictive use of TCA in PwD (Deuschl and Maier).

Previous studies have examined the dispensing of antidepressant drugs using either primary databases or claims data (Arbus et al., 2010; Majic et al., 2010; Rapp et al., 2010; Wetzels et al., 2011; Martinez et al., 2013; Taipale et al., 2014; Giebel et al., 2015; Laitinen et al., 2015; Booker et al., 2016; Breining et al., 2016; David et al., 2016; Jacob et al., 2017; Jobski et al., 2017; Puranen et al., 2017). However, the specific dispensing of TCAs have not yet been analyzed using claims data of the German healthcare system. This information about TCA dispensing behavior would be important for health care providers regarding patients’ safety and guideline-adherent pharmacotherapy (Holt et al., 2010).

The aim of our study was therefore to evaluate the frequency of TCA dispensing over a period of 1 year. In particular, this analysis illustrates how often TCAs were dispensed to PwD over a period of 12 months by evaluating the number of quarters in 2015 with at least one TCA dispensing per patient. Second, we included PwD who were diagnosed with dementia for the first time in the previous year (2014), which allowed us to detect modifications of TCA dispensing during those 12 months after the incident diagnosis of dementia. We hypothesized that physicians avoid dispensing TCAs after a dementia diagnosis owing to their adherence to current guidelines. To the best of our knowledge, this is the first evaluation using claims data to analyze the dispensing of TCAs for PwD in Germany.

## Patients and Methods

For this observational cohort study, we used anonymized claims data from the years 2014 and 2015, provided by a large German statutory health insurance fund (Allgemeine Ortskrankenkasse Niedersachsen, AOK). The local research ethics committee at the University Medical Center Goettingen, Germany, confirmed that the project is exempt from the requirement of a regular review by the committee because all data were anonymized.

We included patients who were diagnosed with dementia for the first time in 2014, based on diagnostic criteria of the International Classification of Diseases (ICD-10-GM codes F00.0, F00.1, F00.2, F00.9, F01.0, F01.1, F01.2, F01.3, F01.8, F01.9, F02.0, F02.3, F03, G30.0, G30.1, G30.8, G30.9, G31.0, G31.82) (International Classification of Diseases, 2018). To be eligible, patients had i) to be ≥65 years at the beginning of 2014, ii) to be continuously insured in 2014 and 2015, and iii) no diagnosis of dementia in the year before the new diagnosis. To confirm a subsequent diagnosis of dementia in the dataset, dementia had to be encoded again at least once (inpatient main or secondary diagnosis) or twice in two different quarters (confirmed outpatient diagnosis) over a period of 12 months after the first codification (Lange et al., 2015). TCAs were identified in the claims data according to the Anatomical Therapeutic Chemical Classification.

We evaluated the frequency of TCA dispensing in 2015 according to the prescription dates. For this 12-month observation period, we examined the total number of patients treated with at least one TCA dispensing. Additionally, we evaluated for how many quarters of 2015 (one, two, three, or all four quarters) a TCA dispensing was registered for each patient and which specialist dispensed the TCA. Furthermore, the dispensing of TCAs before and after the diagnosis of dementia was analyzed. Patients who were first diagnosed with dementia either in the third or the fourth quarter of 2014 were selected, and the dispensing of TCAs was evaluated two quarters before and four quarters after the incident diagnosis. A detailed description of the methods applied is given in a recently published study (Hessmann et al., 2018).

This study aimed at descriptively analyzing dementia patients’ treatment with TCAs. Data are presented as total numbers of cases and percentages or as means with standard deviations (*SD*), median, minimum, and maximum. All statistical analyses were conducted with Microsoft Office Excel 2010 (Microsoft Corporation, Redmond, USA) and SPSS Version 24.0 (IBM SPSS Statistics, Armonk, USA). Significance was defined as α = 0.05, and the normal distribution was assessed with the Kolmogorov–Smirnov test before conducting bivariate analyses. Friedman tests and Cochran’s *Q* tests were used to examine whether TCA dispensing differs before and after diagnosis of dementia.

## Results

**Figure 1:**
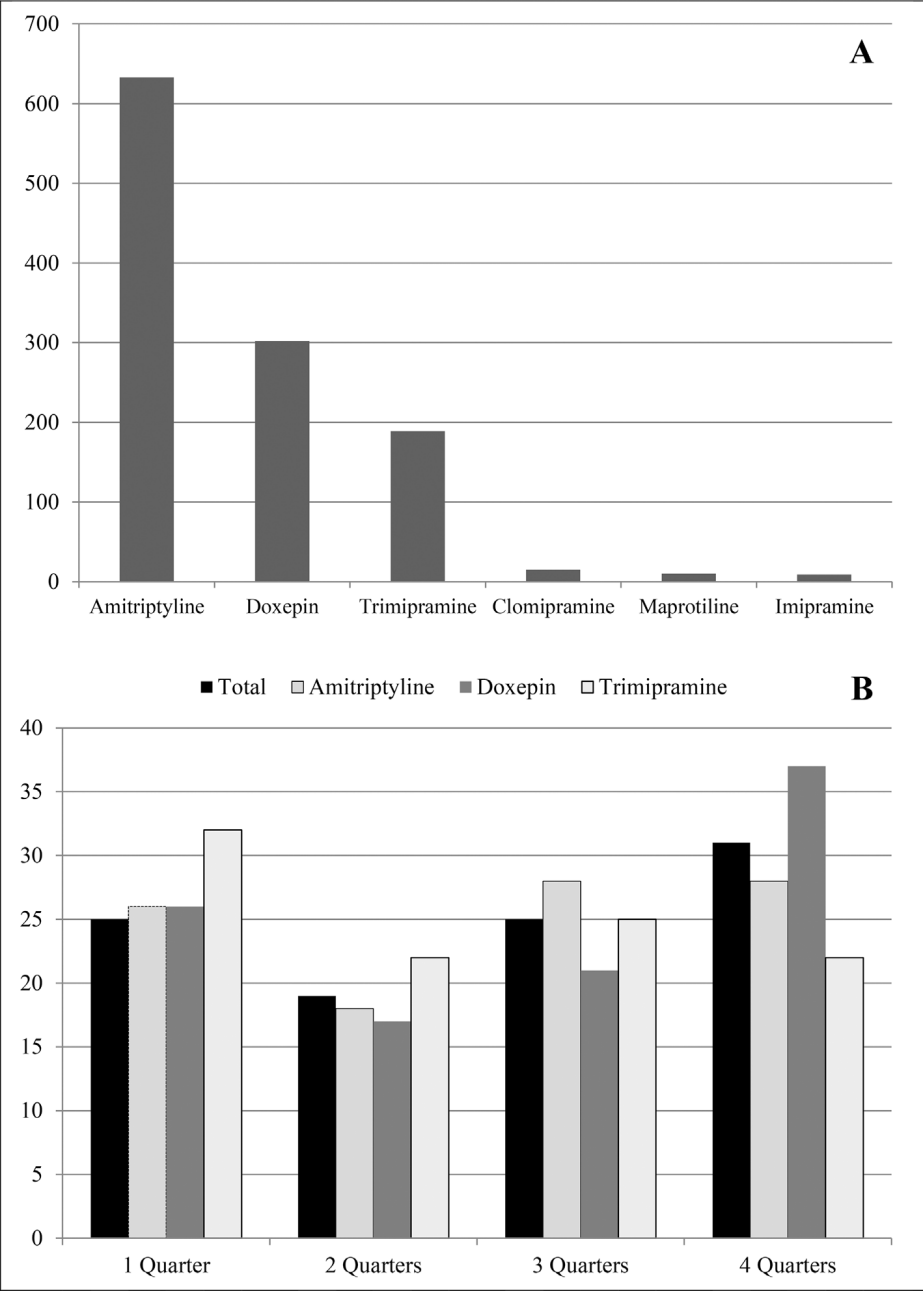
Total number of patients with at least one dispensing of tricyclic antidepressants (TCA) in 2015 **(A)** and frequency (%) of TCA dispensing in 2015 **(B)**.

The study sample was derived from a cohort of 23,232 persons who were registered as incident PwD for the year 2014 in claims data of the AOK Niedersachsen. We excluded 2,875 patients who were below 65 years of age at the beginning of 2014 and/or who were not constantly insured during 2014 and 2015. The remaining 1,125 participants (5.5%) had at least one TCA dispensing in 2015 (77.4% females, *n* = 871) and were 80.5 ± 6.9 years (*median* = 80.0). The majority (75.6%, *n* = 851) had already received a TCA in 2014, while for 24.4%, the first TCA dispensing was encoded in 2015. Depressive syndromes (monophasic or recurrent) were encoded for 764 patients (67.9%) in our cohort. As shown in **Figure 1A**, patients were most often treated with amitriptyline (56.3%, *n* = 633), doxepin (26.8%, *n* = 302), and trimipramine (16.8%, *n* = 189).

In 2015, patients had 3.7 ± 2.6 (*median* = 3.0) dispensings of TCA on average, with an average of 4.0 ± 3.2 (*median* = 3.0) dispensings for doxepin, while patients with amitriptyline received 3.5 ± 2.3 (*median* = 3.0) and those with trimipramine received 3.0 ± 2.0 (*median* = 3.0) dispensings. Of the total 4,914 TCA dispensings in 2015, most dispensings were made by general practitioners (*n* = 3,336, 67.9%), while specialists in internal medicine were responsible for 822 (16.7%) dispensings, and psychiatrists and neurologists for 660 (13.4%).

Next, we examined the number of quarters in 2015 with at least one TCA dispensing per patient (**Figure 1B**). Totally, 31.0% (*n* = 349) of the patients received a dispensing in all four quarters, while 24.9% (*n* = 281) patients received a TCA in one or three quarters, and 19.0% (*n* = 214) had dispensings in two quarters. Most patients treated with trimipramine (32.1%, *n* = 61) received dispensings in only one quarter, while doxepin was dispensed over all four quarters for the majority (37.1%, *n* = 112). However, dispensings in two quarters were less often seen, especially in the case of amitriptyline (18.0%, *n* = 114).

Finally, we evaluated whether the dispensing of TCAs was associated with the incident diagnosis of dementia. We conducted subgroup analyses in those patients with an incident diagnosis in the third or fourth quarter of 2014 who were already treated with TCAs over a period of two quarters prior to the incident diagnosis (30.1%, *n* = 339). Specifically, TCA dispensings among the selected patients were evaluated for two quarters before and four quarters after the incident dementia diagnosis. The number of patients receiving TCAs diminished following the incident diagnosis, although this was not significant, and no differences in the dispensing frequencies of TCAs were seen. A distinguished analysis of the dispensing of each substance also showed no trend towards a diminished dispensing of TCAs, except for doxepin (**Table 1**). However, after adjusting for multiple testing differences in the dispensing of doxepin did not remain significant.

**Table 1:** Dispensing of tricyclic antidepressants (TCA) over two quarters before and four quarters after the diagnosis of dementia in Q3 or Q4 of 2014 (*n* = 339).

	Two quarters before Dx	One quarter before Dx	Quarter of Dx	One quarter after Dx	Two quarters after Dx	Three quarters after Dx	*p* value
**TCA total** (*n*, %) Mean ± SD Median (range)	271 (79.9) 1.50 ± 0.89 1 (1–7)	275 (81.1) 1.42 ± 0.75 1 (1–7)	266 (78.5) 1.54 ± 0.85 1 (1–7)	264 (77.9) 1.44 ± 0.87 1 (1–8)	259 (76.4) 1.43 ± 0.80 1 (1–7)	254 (74.9) 1.37 ± 0.72 1 (1–7)	*p* = 0.305 *p* = 0.090
**Amitriptyline** (*n*, %) Mean ± SD Median (range)	150 (44.2) 1.43 ± 0.79 1 (1–5)	150 (44.2) 1.46 ± 0.72 1 (1–4)	152 (44.8) 1.46 ± 0.75 1 (1–4)	151 (44.5) 1.40 ± 0.73 1 (1–5)	153 (45.1) 1.34 ± 0.64 1 (1–4)	147 (43.4) 1.29 ± 0.62 1 (1–4)	*p* = 0.981 *p* = 0.178
**Doxepin** (*n*, %) Mean ± SD Median (range)	84 (24.8) 1.53 ± 0.97 1 (1–7)	75 (22.1) 1.45 ± 0.91 1 (1–7)	70 (20.6) 1.63 ± 1.07 1 (1–7)	72 (21.2) 1.56 ± 1.16 1 (1–8)	61 (18.0) 1.62 ± 1.14 1 (1–7)	62 (18.3) 1.50 ± 0.90 1 (1–7)	*p* = 0.016 *p* = 0.585
**Trimipramine** (*n*, %) Mean ± SD Median (range)	40 (11.8) 1.48 ± 0.59 1 (1–3)	41 (12.1) 1.22 ± 0.53 1 (1–3)	37 (10.9) 1.46 ± 0.73 1 (1–3)	34 (10.0) 1.26 ± 0.57 1 (1–3)	38 (11.2) 1.37 ± 0.59 1 (1–3)	38 (11.2) 1.32 ± 0.62 1 (1–3)	*p* = 0.667 *p* = 0.257

TCA total (n, %), Number of patients with at least one dispensing of TCAs in the respective quarter; Mean ± SD, Average number of TCA dispensings in the respective quarter; Median (Range), Median number of TCA dispensings in the respective quarter; Quarter of Dx, Quarter 3 or 4 in 2014 when dementia was diagnosed; SD, standard deviation; p-value according to Friedman-tests and Cochran’s Q-tests.

## Discussion

In addition to earlier studies exploring the use of antidepressant drugs among PwD in general (Martinez et al., 2013; Taipale et al., 2014; David et al., 2016; Jacob et al., 2017), this paper specifically focuses on the dispensing of TCA in PwD based on German healthcare claims data. In our study cohort, 5.5% of those patients who were diagnosed with dementia for the first time in 2014 were treated with TCA, while about one third of TCA users did not have a diagnosis of depression. According to international guidelines, TCA should be avoided in PwD due to the risk of anticholinergic side effects (Deuschl and Maier). Therefore, the results of our study underline the importance of a well-considered pharmaceutical treatment of PwD. Additionally, the appropriate implementation of guidelines for the treatment of PwD in healthcare institutions should be further evaluated.

Generally, the use of different databases (primary *vs*. claims data), the included types of dementia, and divergent sample sizes complicate comparisons with earlier studies. Nevertheless, earlier studies in Germany reported antidepressant dispensing rates of about 19 to 47% in PwD (Majic et al., 2010; Giebel et al., 2015). These findings are comparable with European studies determining utility rates of antidepressants of 13 to 40% (Laitinen et al., 2015; Breining et al., 2016). However, the use of TCA was not explicitly evaluated in the majority of earlier studies. Therefore, further studies on the use of TCA would be relevant to assess whether a dispensing rate of 5.5% in PwD is comparable to other samples. In case, further studies report that considerably lower dispensing rates would be highly important to evaluate reasons for a lower use of TCA. Implementing methods to diminish the use of TCA could contribute to the patients’ safety and guideline-adherent treatment.

In our study, TCAs were dispensed most often by general practitioners. This contradicts other studies which showed that PwD who were seen by specialists (psychiatrists or neurologists) had a considerably higher chance of being treated with antidepressants in general (Rapp et al., 2010; Hessmann et al., 2018).

In Germany, claims data do not contain details on indications for the dispensing of certain substances. Our data therefore do not allow for direct conclusions about the appropriateness of TCA dispensing regarding different indications such as depression, anxiety disorders, sleep disturbances, or chronic pain syndrome, but it can provide clues on potentially inappropriate use of TCAs. In a prospective study, Wetzels et al. reported that more than 60% of PwD living in nursing homes received antidepressants over a period of 2 years, although depressive symptoms were not observed for many of these patients during clinical examination (Wetzels et al., 2011). However, some authors also suggest that antidepressants may be under-used in PwD. Giebel et al. showed that less than half of all PwD with clinically relevant depression received antidepressant drugs (Giebel et al., 2015).

The specific indications for TCA in PwD should be evaluated in future studies to understand the medical background of the dispensing. For this purpose, the attending physicians should be directly involved, e.g., using standardized questionnaires or qualitative interviews. Nevertheless, the relatively high number of PwD with a comorbid depression of about 67% might be the most likely reason for the dispensing of TCAs. Furthermore, the frequency of dispensing varied between the different TCAs. For example, trimipramine was dispensed in only one quarter during the study period by majority. This might be associated with the common use of trimipramine for sleeping disorders which do not necessarily have to be treated permanently.

## Limitations

Although the effective study sample consisted of more than 20,000 patients, our data do not cover the dispensing of TCAs for the entire German population of PwD. Another limitation concerns the diagnostic codes in claims data which are encoded. In particular, only those medical conditions fulfilling the diagnostic criteria according to the ICD-10-GM (e.g., major depression) are registered by the attending physicians. In contrast, disorders not fulfilling these criteria are usually not encoded. This is especially important for the evaluation of indications for TCAs, which are also dispensed as symptomatic treatment for certain psychiatric disturbances (e.g., sleep disorders, anxiety). Therefore, drawing conclusions about the appropriateness of a TCA dispensing based on claims data is generally limited and additional clinical information is required. In addition, more detailed analyses on the number of dispensings as well as the defined daily doses in each quarter are planned on the basis of these claims data.

## Author Contributions

PH, JZ, JS, SE, and BK contributed to the study design, the data analysis, and the writing of the manuscript. JV, MA-H, CW-M, and JW contributed to the data interpretation and critically reviewed the manuscript.

## Conflict of Interest Statement

Anonymized data were provided by the AOK Niedersachsen. JW is supported by an Ilídio Pinho professorship and iBiMED (UID/BIM/04501/2013), at the University of Alveiro, Portugal. JW received honoraria for consulting activities, lectures or advisory board participation from Pfizer, Eli Lilly, Hoffmann-La-Roche, MSD Sharp + Dome, Janssen-Cilag GmbH, Immungenetics AG, Boehringer Ingelheim. CW-M cooperates with LivaNova GmbH, Janssen-Cilag GmbH, Servier GmbH, Vitos Clinics, Privatinstitut für Klinikmanagement, University of Heidelberg, Deutsches Krankenhausinstitut, Deutsche Krankenhausgesellschaft. PH was financially supported by a scholarship from the Research School for Translational Medicine at the University Medical Center in Goettingen (Göttinger Kolleg für Translationale Medizin), which was funded by the Lower Saxony Ministry of Science and Culture (Niedersächsisches Ministerium für Wissenschaft und Kultur). 

The remaining authors declare that the research was conducted in the absence of any commercial or financial relationships that could be construed as a potential conflict of interest.
